# Metachronous Intracranial Germinoma Recurrence After Long-Term Remission: A Case Report

**DOI:** 10.7759/cureus.113793

**Published:** 2026-08-01

**Authors:** Ariadna Perea Gill, Jesús Cienfuegos-Meza, Vanessa A De Aguas, Yenney Reyes Pimentel, Jazmín M Tapia Cárdenas, Tania J Cordova Rodas, Alejandro Miranda-Gonzalez, Mónica Alicia Sierra del Río, Carlos Valladares, Sara Patricia Perez Reyes

**Affiliations:** 1 Medicine, University of Guanajuato, Guanajuato, MEX; 2 Neuropathology, Instituto Nacional de Neurología y Neurocirugía Manuel Velasco Suárez, Mexico City, MEX; 3 Medicine, Faculty of Health Sciences, Simón Bolívar University, Barranquilla, COL; 4 Medicine, National Autonomous University of Mexico, Mexico City, MEX; 5 Medicine, Catholic University of Cuenca, Cuenca, ECU; 6 Neurosciences, Spine Surgery Service, Hospital Regional de Alta Especialidad del Bajío, León, MEX; 7 Neuro-Oncology, Hospital Regional de Alta Especialidad del Bajío, León, MEX; 8 Pulmonary and Critical Care, University of Miami Miller School of Medicine, Miami, USA

**Keywords:** intracranial germinoma, late recurrence, metachronous tumor, pineal region, tumor markers

## Abstract

Intracranial germinomas are rare central nervous system neoplasms. Their diagnosis relies on clinicoradiological correlation and tumor marker assessment. Radiotherapy alone often achieves complete remission; however, late recurrences in atypical locations have been reported, underscoring the importance of timely diagnosis, appropriate treatment, and long-term surveillance in patients with intracranial germinoma. We report the case of a 22-year-old man who presented with severe pulsatile headache, photophobia, phonophobia, vomiting, bilateral thigh weakness, and urinary incontinence. Computed tomography revealed a pineal tumor with obstructive hydrocephalus, prompting ventriculoperitoneal shunt placement. Tumor markers were elevated (serum beta human chorionic gonadotropin (β-hCG) 18.46 mIU/mL, cerebrospinal fluid (CSF) β-hCG 16.8 mIU/mL, serum alpha-fetoprotein (AFP) 2.71 ng/mL, and CSF AFP 0.2 ng/mL). Radiotherapy achieved complete remission; however, the patient subsequently developed radiation-induced hypopituitarism requiring lifelong hormone replacement therapy. Thirteen years after the initial diagnosis, surveillance imaging demonstrated periventricular and callosal enhancement associated with mildly elevated tumor markers. Biopsy revealed OCT4 and CD117 positivity, confirming a new germinoma interpreted as a metachronous neoplasm. This case highlights the value of comprehensive diagnostic evaluation and emphasizes the need for long-term, multidisciplinary follow-up due to the risk of late sequelae and atypical recurrences in patients with intracranial germinoma.

## Introduction

Central nervous system (CNS) germ cell tumors are rare neoplasms arising from primordial germ cells that fail to complete their embryonic migration to the developing gonads, allowing ectopic germ cells to persist within the CNS. They account for less than 0.5% of primary intracranial tumors in Western countries [1]. Germinomas account for approximately 3% of pediatric brain tumors, with nearly 90% diagnosed before the age of 20 [2].

These tumors show a marked predilection for midline structures, particularly the pineal and suprasellar regions, which explains their frequent association with obstructive hydrocephalus and intracranial hypertension [3]. Clinical presentation depends on tumor location and mass effect and commonly includes headache, vomiting, visual disturbances, and symptoms of hypothalamic-pituitary dysfunction such as diabetes insipidus, central hypothyroidism, and hypogonadism [4].

Diagnosis requires integration of clinical findings, neuroimaging, and serum and cerebrospinal fluid (CSF) tumor markers. Although imaging and tumor marker profiles may strongly suggest the diagnosis, histopathological confirmation remains necessary when these findings are inconclusive, with immunohistochemical markers such as OCT4 and CD117 supporting the diagnosis [2,5,6].

Because of their radiosensitivity, germinomas are associated with excellent long-term survival following contemporary treatment strategies [7]. Distinguishing recurrent disease from a metachronous germinoma is clinically important. Whereas recurrence reflects the reappearance of previously treated disease, a metachronous germinoma refers to the development of a new germ cell tumor after a prolonged disease-free interval. This distinction underscores the importance of prolonged surveillance in patients with CNS germinomas [7].

Despite their favorable prognosis, metachronous germ cell tumors developing after prolonged disease-free intervals remain exceedingly rare. We report a patient who developed a metachronous germinoma more than a decade after complete remission of a pineal germinoma, highlighting an exceptionally uncommon pattern of disease evolution and reinforcing the importance of prolonged surveillance, even among patients with initially favorable outcomes.

## Case presentation

In 2010, a 22-year-old man with a history of tobacco use beginning at age 12 (approximately one pack per month) and episodic alcohol consumption starting at age 13 presented to the neurosurgery department with a two-month history of severe holocranial, pulsatile headache, rated 10/10 in intensity. The headache was associated with photophobia, phonophobia, nausea, vomiting, and a two-day history of diplopia, raising suspicion for intracranial hypertension.

On examination, he was conscious, oriented, and cooperative. Vital signs were within normal limits. Neurological status was assessed using the Glasgow Coma Scale (GCS). Neurological assessment revealed a GCS score of 15, isochoric and reactive pupils, intact cranial nerves, and no motor or sensory deficits at the time of evaluation [8]. However, the patient reported recent episodes of lower extremity weakness and urinary incontinence. Given the clinical picture of intracranial hypertension and diplopia, computed tomography was performed, revealing a pineal mass with obstructive hydrocephalus. A ventriculoperitoneal shunt was placed, and CSF analysis was obtained (Table 1).

**Table 1:** Serum and CSF tumor marker analysis Reference ranges may vary according to laboratory standards CSF: cerebrospinal fluid; β-hCG: beta-human chorionic gonadotropin

Sample	β-hCG (mUI/mL)	Reference range β-hCG (mUI/mL)	Alpha-fetoprotein (ng/mL)	Reference range of alpha-fetoprotein (ng/mL)
Serum	18.46	<5	2.71	<10
CSF	16.8	Undetectable	0.2	Undetectable

The diagnosis of pineal germinoma was established based on the integration of clinical, radiological, and biochemical findings. Clinically, the patient presented with symptoms of intracranial hypertension, including severe headache, vomiting, and diplopia. Radiological evaluation demonstrated a pineal region mass causing obstructive hydrocephalus, a characteristic location for intracranial germ cell tumors. Biochemically, elevated serum and CSF beta-human chorionic gonadotropin (β-hCG) levels with normal alpha-fetoprotein (AFP) values further supported the diagnosis of germinoma.

The patient underwent fractionated external-beam radiotherapy (33 sessions) in 2010, achieving a complete clinical and radiological response. He was followed with serial serum and CSF tumor markers and periodic magnetic resonance imaging (MRI), with no evidence of recurrence during the first 12 years (Table 2).

**Table 2:** CSF analysis results during follow-up Reference ranges: serum β-hCG < 5 mIU/mL; CSF β-hCG undetectable; serum AFP < 10 ng/mL; CSF AFP undetectable Reference ranges may vary according to laboratory standards CSF: cerebrospinal fluid; β-hCG: beta-human chorionic gonadotropin; AFP: alpha-fetoprotein

Time since treatment	Serum β-hCG (mUI/mL)	Serum alpha-fetoprotein (ng/mL)	CSF β-hCG (mUI/mL)	CSF AFP (ng/mL)
1 year	1.05	4.36	1.2	0.2
2 years	0.5	2.86	<0.5	0.2

**Figure 1:**
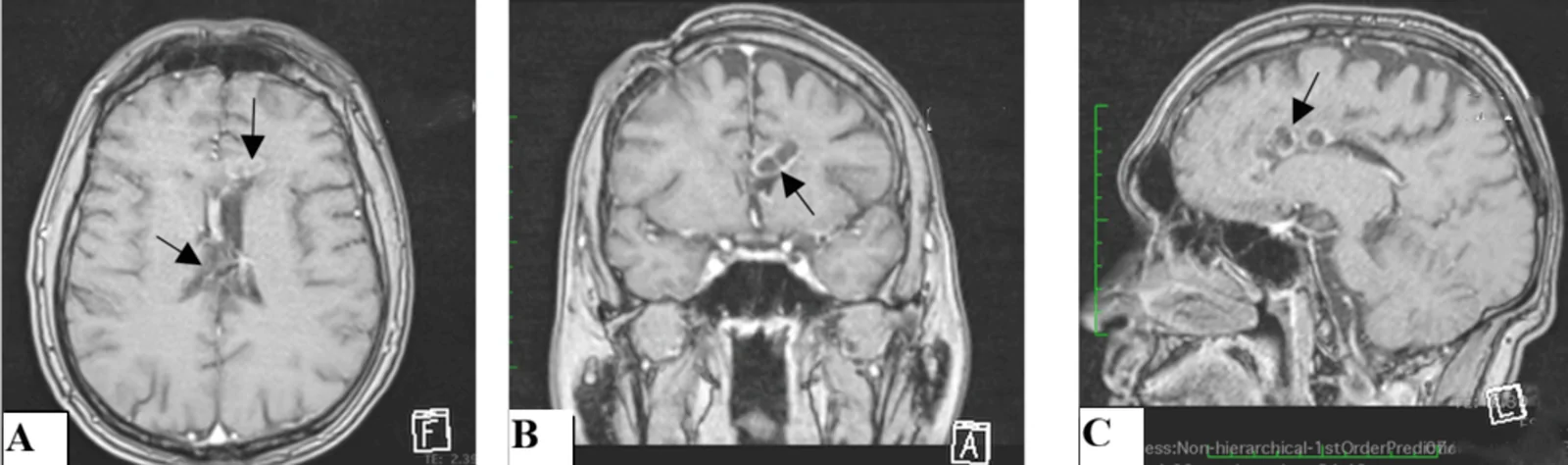
MRI findings (A) Axial T1-weighted contrast-enhanced brain MRI. A midline 87 lesion involving the ventricular wall is observed, showing an irregular morphology with 88 heterogeneous gadolinium enhancement (black arrows). (B) Coronal T1-weighted contrast-enhanced 89 brain MRI (black arrow). (C) Sagittal T1-weighted contrast-enhanced brain MRI (black arrow) MRI: magnetic resonance imaging

In 2019, the patient developed radiation-induced hypopituitarism, requiring lifelong hormone replacement therapy with levothyroxine, prednisone, desmopressin, growth hormone, and testosterone. In 2023, follow-up MRI demonstrated irregular, nodular, heterogeneous periventricular and ependymal enhancement involving the corpus callosum (Figure 1), consistent with disease recurrence. Tumor marker levels at that time are presented in Table 3, and CSF cytology was negative for malignant cells.

**Table 3:** CSF analysis results at tumor recurrence Reference ranges: serum β-hCG < 5 mIU/mL; CSF β-hCG undetectable; serum AFP < 10 ng/mL; CSF AFP undetectable CSF was negative for neoplastic cells Reference ranges may vary according to laboratory standards CSF: cerebrospinal fluid; β-hCG: beta-human chorionic gonadotropin; AFP: alpha-fetoprotein

Sample	β-hCG (mUI/mL)	Alpha-fetoprotein (ng/mL)
Serum	<1	4.54
CSF	2.72	0.01

Over subsequent months, the patient experienced progressive decline characterized by memory impairment, inattention, disconnection from his surroundings, photophobia, phonophobia, episodic moderate headache, gait instability, and worsening hydrocephalus requiring shunt revision. CSF studies at that time remained normal.

**Figure 2:**
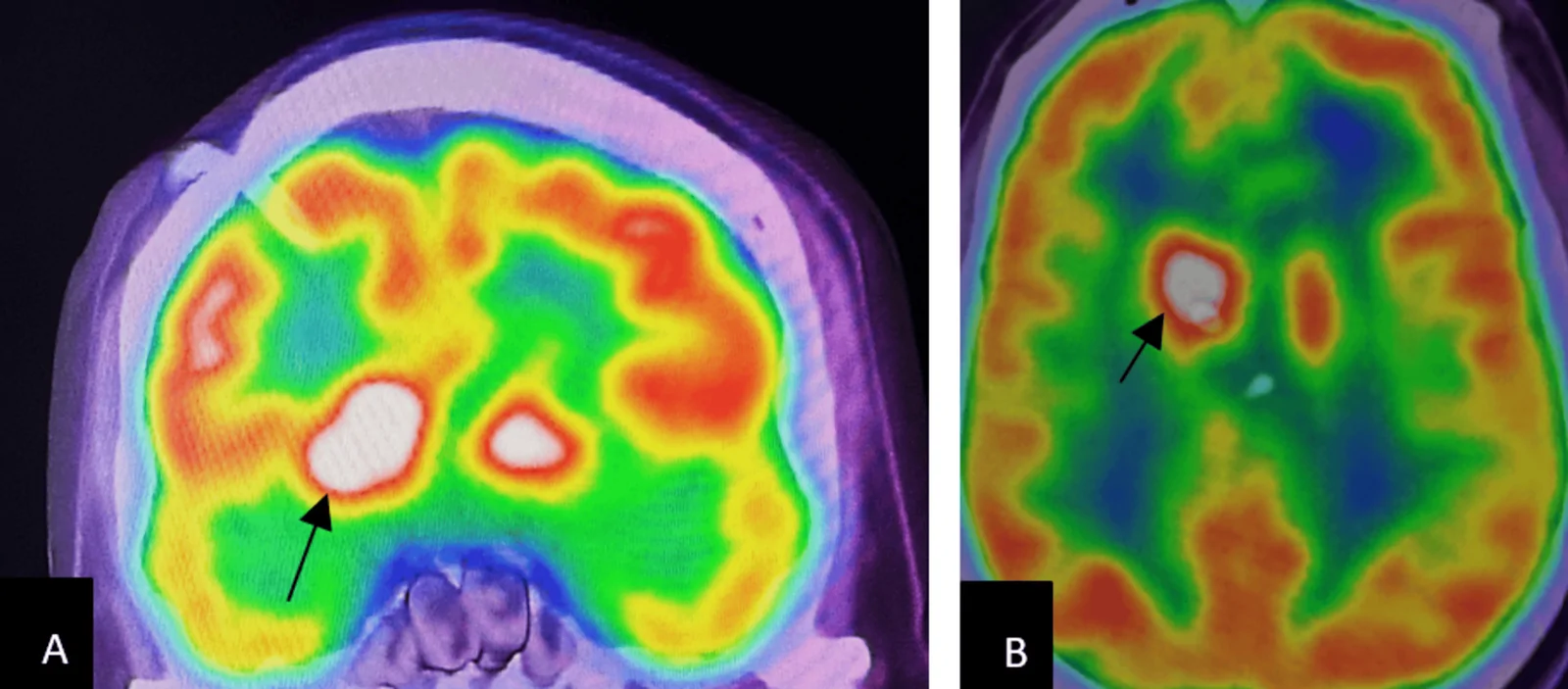
PET findings (A) PET coronal FDG-PET scan demonstrating marked SVUmax 10.5 hypermetabolism in the periventricular regions and basal ganglia (black arrow). (B) Axial FDG-PET hypermetabolism in the periventricular regions (black arrow) FDG-PET: fluorodeoxyglucose positron emission tomography

In 2023, the positron emission tomography (PET) imaging revealed increased metabolic activity in the right periventricular region at the occipital horn of the lateral ventricle and in the left centrum semiovale. Given concern for primary CNS lymphoma, a biopsy was performed via interhemispheric craniotomy. PET imaging revealed increased metabolic activity in the right periventricular region at the occipital horn of the lateral ventricle and in the left centrum semiovale (Figure 2).

**Figure 3:**
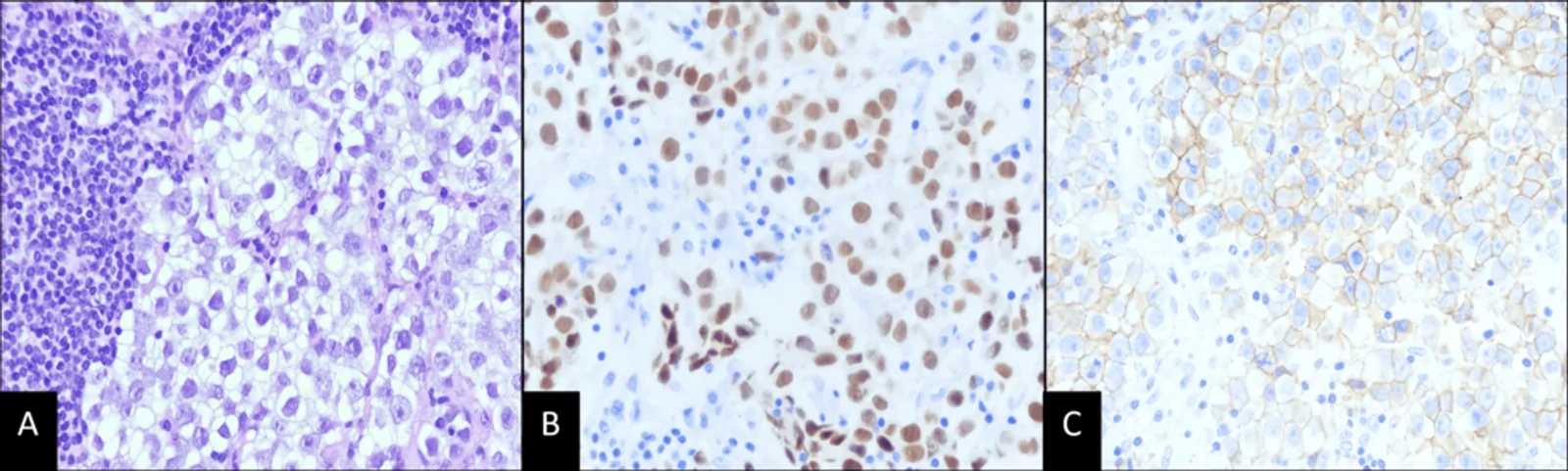
Histopathological findings of cerebral germinoma (A) Higher magnification of plump neoplastic cells with round nuclei, prominent nucleoli, and clear cytoplasm. Lymphoid aggregates surround neoplastic cells (H&E; 40×). (B) Neoplastic cells are immunoreactive to OCT3/4 (40×). (C) Diffuse cytoplasmic membrane immunoreactivity to CD117 (40×) H&E: hematoxylin and eosin

Because primary CNS lymphoma was considered in the differential diagnosis, an interhemispheric craniotomy with biopsy was performed in 2024. Histopathology showed a malignant germ cell neoplasm consistent with germinoma. The tumor was arranged in solid nests partially bounded by thin fibrovascular septa with a prominent perivascular lymphocytic infiltrate (Figure 3A). The neoplastic cells were medium-sized and ovoid, with delicate retracted cytoplasm and nuclei showing vesicular chromatin, some with distinct nucleoli, along with both typical and atypical mitotic figures. Higher magnification revealed plump neoplastic cells with round nuclei, prominent nucleoli, and clear cytoplasm surrounded by lymphoid aggregates (Figure 3A). Multinucleated giant cells with syncytiotrophoblastic features were also identified, with focal necrosis involving less than 5% of the examined fields (Figure 2B). Immunohistochemistry demonstrated strong nuclear OCT3/4 immunoreactivity (Figure 3B) and diffuse membranous CD117 positivity (Figure 3C).

Considering the initial pineal germinoma, the complete clinical and radiological remission achieved after treatment, the prolonged disease-free interval of 13 years, and the absence of residual or recurrent disease at the primary pineal site, the newly identified lesion was interpreted as a metachronous germ cell neoplasm of the same histologic lineage arising in the periventricular region rather than persistent or locally recurrent disease. Its distinct anatomical location, together with the long latency period following successful treatment, supports the diagnosis of a metachronous intracranial germinoma.

## Discussion

CNS germinomas typically arise in midline structures and occur predominantly in young patients. Despite their malignant nature, they demonstrate remarkable radiosensitivity, achieving disease control rates exceeding 90% with current radiotherapy protocols [9]. Their predilection for the pineal and suprasellar regions explains the high incidence of obstructive hydrocephalus and intracranial hypertension at presentation [10].

Although overall prognosis is excellent, a relevant proportion of patients develop late complications, particularly endocrine dysfunction such as hypopituitarism and growth hormone deficiency related to irradiation of the hypothalamic-pituitary axis. Neurocognitive sequelae have also been reported, supporting the need for long-term, multidisciplinary follow-up [5,7].

Unlike many other intracranial tumors, germinomas are frequently managed without upfront surgical resection because of their characteristic radiological appearance, supportive tumor marker profile, deep midline location, and marked radiosensitivity. Surgical intervention is generally reserved for CSF diversion in patients with hydrocephalus, tissue diagnosis when the diagnosis is uncertain, or management of residual or recurrent disease. This approach has achieved excellent long-term disease control while minimizing the morbidity associated with surgery in deep-seated lesions [2,9].

Although most recurrences occur within the first several years after treatment, late recurrences have been reported, underscoring the importance of lifelong vigilance [7]. The uniqueness of this case lies not only in the prolonged disease-free interval but also in the development of a metachronous periventricular germinoma without radiological evidence of residual or recurrent disease at the primary pineal site.

Published reports indicate that germinomas typically recur within previously irradiated fields or within the same ventricular compartment. Recurrences arising in distant, previously uninvolved sites are far less common and almost never appear beyond 10 years of follow-up [7]. Thus, the emergence of a new periventricular mass 13 years after initial diagnosis and following a prolonged tumor-free interval represents one of the most atypical patterns described for this disease.

Additionally, tumor markers showed only mild β-hCG and AFP elevations, and CSF cytology remained negative. This biochemical profile favors the diagnosis of a new lesion of identical cellular lineage rather than a late recurrence of the original tumor. Metachronous germ cell tumors, defined as new germ cell neoplasms arising after a prolonged disease-free interval, are exceptionally rare and generally occur much earlier than in this case.

Diagnosis in this patient required integrating clinical symptoms, neuroimaging, and biomarker analysis. The heterogeneous enhancement pattern and periventricular distribution on MRI broadened the differential diagnosis, which included primary CNS lymphoma, high-grade glioma, metastatic disease, ependymal tumors, inflammatory or demyelinating lesions, and recurrent germinoma. Among these entities, primary CNS lymphoma was considered the leading alternative diagnosis because of the lesion's deep periventricular location and marked metabolic activity on PET. In the absence of a definitive radiologic pattern and given the nonspecific tumor marker profile, histopathological examination was essential to establish the diagnosis.

Histopathologic examination demonstrated classic germinoma features, including nests of tumor cells separated by thin fibrovascular septa and prominent perivascular lymphocytic infiltrates [6]. Immunohistochemical positivity for OCT4 and CD117 further supported the diagnosis, as both markers are sensitive and specific for germinomatous tumors. The presence of multinucleated syncytiotrophoblastic giant cells accounted for the modest elevation in β-hCG [11].

This case emphasizes the need for continued long-term surveillance, even after complete remission, due to the possibility, albeit low, of metachronous germ cell neoplasms. It also highlights the importance of comprehensive diagnostic evaluation when imaging or clinical presentation is atypical.

## Conclusions

This case illustrates the typical initial course of a pineal germinoma while emphasizing that, even after complete response to radiotherapy, patients remain at risk for late endocrine sequelae and, in rare cases, metachronous germ cell tumors. The development of a new lesion in a distinct anatomical location more than a decade after initial treatment highlights the limitations of short-term surveillance strategies. In CNS germinomas, long periods of clinical and radiological stability do not exclude the possibility of late tumor emergence. Therefore, prolonged, multidisciplinary follow-up with sustained clinical vigilance is essential, particularly when new neurological or radiological changes arise, regardless of the time elapsed since primary therapy.
